# Exploring young women’s menstruation-related challenges in Uttar Pradesh, India, using the socio-ecological framework

**DOI:** 10.1080/26410397.2020.1749342

**Published:** 2020-04-20

**Authors:** Ellen McCammon, Suchi Bansal, Luciana E. Hebert, Shirley Yan, Alicia Menendez, Melissa Gilliam

**Affiliations:** a Research Specialist, Center for Interdisciplinary Inquiry and Innovation in Sexual and Reproductive Health at the University of Chicago, Chicago, IL, USA; b Research Associate, Harris School of Public Policy, University of Chicago, Chicago, IL, USA; c Ellen H. Block Professor of Health Justice, Center for Interdisciplinary Inquiry and Innovation in Sexual and Reproductive Health at the University of Chicago, Chicago, IL, USA

**Keywords:** Adolescent health, youth health, gender inequality, global health, life course interviews, menarche, menstrual stigma, gender norms, young people

## Abstract

Menstruation frequently poses psychological, social, and health challenges for young women living in low- and middle-income countries. In countries such as India, where menstruation is stigmatised, it can be particularly difficult. This paper examines challenges related to menstruation for young women living in slums in Lucknow, Uttar Pradesh, India. The research was informed by the socio-ecological model. Life course interviews were conducted with 70 young women ages 15–24 living in the slums of Lucknow. Thematic analysis was used to identify salient themes regarding individual, social, and systemic challenges related to menstruation. On the individual level, young women lack knowledge about menstruation. In the social sphere, young women experience stigma around menstruation, lack opportunities to discuss menstruation, and experience limitations around mobility and other activities during menstruation. At the institutional level, for example in school, there are few resources to support menstruating young women as toilets are dirty and doors are broken. Therefore, menstruating adolescents and young women in Lucknow, Uttar Pradesh, India, face an array of challenges at multiple levels. These findings suggest that multi-level interventions are warranted to create a supportive context for menstruation.

## Résumé

La menstruation pose fréquemment des problèmes psychologiques, sanitaires et sociaux aux jeunes femmes vivant dans des pays à revenu faible ou intermédiaire. Dans des pays comme l’Inde, où la menstruation est cause de stigmatisation, ces problèmes peuvent être particulièrement aigus. L’article examine les obstacles liés à la menstruation pour les jeunes femmes vivant dans des bidonvilles à Lucknow, Uttar Pradesh, Inde. La recherche a été guidée par le modèle socio-écologique. Des entretiens sur leur parcours de vie ont été réalisés avec 70 jeunes femmes âgées de 15 à 24 ans qui vivaient dans des bidonvilles de Lucknow. L’analyse thématique a été utilisée pour identifier les points saillants concernant les problèmes individuels, sociaux et systémiques relatifs à la menstruation. Au niveau individuel, les jeunes femmes manquent de connaissances sur la menstruation. Dans la sphère sociale, elles connaissent la stigmatisation autour de la menstruation, manquent de possibilités d’en parler et se heurtent à des limites autour de la mobilité et d’autres activités pendant la menstruation. Au niveau institutionnel, par exemple à l’école, il y a peu de ressources pour soutenir les femmes qui ont leurs règles car les toilettes sont sales et les portes sont cassées. Par conséquent, les adolescentes et les jeunes femmes qui ont leurs règles à Lucknow, Uttar Pradesh, Inde, font face à un éventail de difficultés à de multiples niveaux. Ces conclusions suggèrent que des interventions à plusieurs niveaux sont nécessaires pour créer un contexte favorable à la menstruation.

## Resumen

La menstruación frecuentemente plantea retos psicológicos, sociales y de salud para las mujeres jóvenes que viven en países de ingresos bajos y medios. En países como India, donde la menstruación es estigmatizada, puede ser particularmente difícil. Este artículo examina los retos relacionados con la menstruación para las jóvenes que viven en barrios marginales de Lucknow, en Uttar Pradesh, India. La investigación fue informada por el modelo socioecológico. Se realizaron entrevistas de trayectoria vital con 70 mujeres jóvenes entre 15 y 24 años que viven en los barrios marginales de Lucknow. Se utilizó el análisis temático para identificar temas destacados relacionados con retos personales, sociales y sistémicos relacionados con la menstruación. A nivel personal, las jóvenes carecían de conocimientos sobre la menstruación. En la esfera social, las jóvenes sufren estigma relacionado con la menstruación, carecen de oportunidades para hablar sobre la menstruación y experimentan limitaciones en torno a su movilidad y otras actividades durante la menstruación. A nivel institucional, por ejemplo en la escuela, existen pocos recursos para apoyar a las jóvenes durante la menstruación, ya que los inodoros están sucios y las puertas rotas. Por ello, las adolescentes y jóvenes menstruantes en Lucknow, Uttar Pradesh, India, enfrentan una variedad de retos en múltiples niveles. Estos hallazgos indican que se necesitan intervenciones en múltiples niveles para crear un contexto de apoyo para las jóvenes durante la menstruación.

## Introduction

The onset of menstruation can be challenging for young women living in low- and middle-income countries.^1^ Many young women lack resources for safely managing menstruation at home and in school, such as private bathrooms with clean water.^2,3^ In some countries, menarche is associated with higher rates of school dropout among young women.^1,4^ School dropout can be due to multiple factors, including social pressure to marry and bear children with the onset of menses^3,4^ and the difficulties of managing menstruation at school.^5–8^ Specific menstrual hygiene management (MHM) challenges faced by young women in schools include: inadequately clean and safe bathrooms, lack of running water and menstrual supplies, and teasing from male peers and teachers about menstruation.^1,6,9^

Education confers numerous health and well-being benefits on young women, including increased access to health care,^10,11^ higher age at marriage and lower fertility rates,^12,13^ and lower risk of experiencing intimate partner violence.^13,14^ Therefore, ensuring that menstruation does not keep young women from attending school is beneficial from both a public health and gender equity perspective.^3,7,15,16^

Menstruation poses particular challenges to young women living in India and other South Asian countries due to high levels of menstrual stigma.^16–19^ Menstruating girls and women are often considered dirty or impure.^3,7,15,16^ Young women face a number of taboos and restrictions on their mobility, daily activities, and practices for managing hygiene during menstruation.^2,3,16,17,20,21^ They may face restrictions on bathing^19,22^ and be limited in their ability to move around inside and outside the home for fear of contaminating others.^17,20,23^ Parents may lack accurate knowledge about MHM, further limiting the support that they can provide.^17,22,24^ Restrictions such as these have clear implications for the health, safety, and comfort of menstruating adolescent girls and young women. To address the difficulties of safely managing menstruation in India, it is important to better understand young women’s experiences with menstruation.

This paper examines the challenges associated with menstruation among young women living in the slums of Lucknow, Uttar Pradesh (UP). Uttar Pradesh (UP) is home to 19.3% of all Indian adolescents ages 10–19 and 17.5% of India’s youth ages 15–24,^25^ suggesting that interventions in this state could be influential for adolescent health in India. Furthermore, UP is a highly gender-inequitable Indian state.^26^ UP is in the lowest quintile of all Indian states for girls’ enrolment in secondary school, and women make up only 16% of the working population, again among the lowest percentages of all Indian states.^26^ UP is also in the bottom tenth of Indian states for female to male sex ratio.^25^ Gender inequity exacerbates young women’s menstruation-related difficulties because women in these settings often lack the power to make household, community, and policy decisions that prioritise menstruation-related needs.^3,27^ Because of the sheer number of young women in UP and its gender inequality, UP is a high priority region for examining young women’s experiences with menstruation.

The socio-ecological model (SEM) was used to frame the menstruation-related challenges of young women living in UP. Ecological models of health promotion like the SEM emerged in the 1980s from earlier social and behavioural sciences theories and have been endorsed by major national and international health promotion organisations as crucial to designing effective health interventions.^28,29^ The SEM conceptualises the many factors influencing health behaviours as a complex system with multiple levels.^30^ The SEM posits that people are affected not just by individual-level factors like their own knowledge and attitudes, but by interpersonal/social, institutional, community, and policy-level factors that shape their specific context. Thus, factors on different levels interact to facilitate or impede a person’s ability to perform health behaviour(s). The SEM further posits that people and their behaviours are embedded in communities in an interdependent fashion, emphasising the complexity and heterogeneity of a given community and the possibility of identifying suitable points for intervention.^28^ The SEM further suggests that the goal of interventions should be to create and maintain a context that facilitates health, or else interventions are likely to be short-lived and unsustainable.^28^ An important first step to designing such interventions is describing the existing context in a given community in relation to the health issue of interest.^28^ The SEM has been used effectively to explore and conceptualise other health and gender equality issues for adolescents in South Asia, including peer violence,^31^ maternal health care,^32^ and intimate partner violence.^33^

## Methods

### Recruitment and data collection

Study activities took place as part of a parent study called Kissa Kahani, a multiphase study examining the lives of young people in urban slums in UP, India. The aim of Kissa Kahani was to identify modifiable factors to improve young people’s sexual and reproductive health and family planning outcomes.

This paper focuses on life course interviews conducted with young people ages 15–24 living in Lucknow slums. Local non-governmental organisations (NGOs) conducted recruitment. While young men were interviewed as part of the project, this paper considers only the interviews conducted with young women.

Consent was obtained for young women 18+; parental consent with youth assent was obtained for participants younger than 18. All participants completed a baseline survey on demographic characteristics. A trained female interviewer from an Indian partner organisation completed interviews in Hindi. Through the life course interview, menarche and menstruation were contextualised within the broader framework of young women’s life trajectories. The interview guide explored significant moments in the lives of youth, including their early childhood, education and school, puberty experiences, and thoughts on being a girl in their family and community. All sessions were audio recorded.

### Data analysis

Recorded interviews were translated into English and transcribed by a third-party translation agency and validated by the India-based project team. Interviews averaged an hour in length. Transcriptions were de-identified and uploaded in Dedoose, a web-based qualitative coding software.^34^

The research team conducted data analysis using an iterative process of thematic analysis.^35^ Team members first familiarised themselves with the dataset as a whole. Then, four members of the research team closely read a subset of the transcripts, independently noted down major descriptive themes, and met to complete a draft codebook. Afterwards, two members of the team used the draft codebook to code the same subset of transcripts, refining codes as necessary. Using the refined codebook, all research team members coded another subset of transcripts and then met to discuss and resolve disagreements. Once researchers ensured sufficient coder agreement, the remaining transcripts were divided up between research team members and coded independently.

Once coding was complete, research team members further analysed the data by creating matrices to distil themes within the codes. By examining the quotations, new subthemes emerged. To further analyse these new sub-themes, each team member wrote a comprehensive memo synthesising and describing the sub-themes. Team members reviewed each memo to validate and further develop findings, and to situate major themes within the socio-ecological model: intrapersonal/individual, interpersonal/social, and institutional. Analysis was limited to the first three levels of the socio-ecological model as the research team intended to identify potential interventions on these levels. The Institutional Review Board (IRB) for the University of Chicago’s Biological Sciences Division and the Sigma IRB in India approved all protocols for this study.

## Results

Seventy interviews were conducted with young women between the months of March and May 2016.

Participants ranged in age from 15 to 24, median age 18. Nearly all participants were unmarried, and 90% of the study sample were Hindu; the remainder were Muslim. Most participants had completed at least some high school, although education levels varied. See Table 1 for more details. Relevant themes surfaced by our analysis are presented below according to the socioecological model to reflect the individual, social, and institutional challenges associated with menstruation faced by young women in the slums of Lucknow.

**Table 1.** Sample characteristics

	Number (*n* *=* 70)	%
**Age (*median age*: 18)**		
15–16	24	34
17–18	20	29
19–21	18	26
22–24	8	11
**Religion**		
Hindu	64	91
Muslim	6	9
**Highest education completed**		
Nursery only	1	1.4
Some primary	10	14
Primary school (8th grade)	8	11
Some high school	21	30
High school	13	19
College or higher	11	16
No answer/ambiguous answer	6	9
**Ever married**	4	6
**Ever given birth**	3	4

### Individual level challenges

The two main individual-level challenges related to menstruation were a lack of knowledge about menstruation and receiving incorrect information.

#### Ignorance about menstruation

Young women reported significant gaps in their knowledge about menstruation. Very few participants reported learning or hearing anything about menstruation prior to menarche, and most participants said they had no knowledge of menstruation before they started menstruating. The majority of participants had a negative emotional response to their initial menstruation, characterising menarche as an unpleasant shock:

“I informed my mother. I was shocked. I went to my mother and shockingly asked her about the blood. I was much tensed. My mother had not informed me that I will get something like that. I did not have any clue about it. I did not know that something like that would happen to me.”

Following menarche, the majority of young women received some form of information or advice on menstruation from female relatives. Nevertheless, they still had knowledge gaps regarding menstruation. Participants had very basic to no understanding of the biology of menstruation, or why it happens. In the words of two participants:

“My mother told me about this [menstruation]. But she didn’t tell me why this happens? If people don’t have it then they go for medical treatment.”

“I know what it is but I didn’t know why and how it happens.”

Thus, most young women were unaware of menstruation before menarche and continued to have minimal knowledge once it occurred.

#### Misinformation about menstruation

When young women participants did learn about menstruation, they often received misinformation and therefore held misconceptions about menstruation. The most common misconception among participants was that eating particular foods would increase menstrual pain or flow. Some believed that their touch could contaminate or cause harm; for example, many participants stated that touching pickles would cause the pickles to spoil. A few were advised to avoid boys during menstruation because they might become pregnant, suggesting possible misconceptions about fertility throughout the menstrual cycle. Misinformation about washing and grooming was relatively common among participants, as in the following example:

“First and second day, should not take bath and but can have bath on third day. I have a friend who had bath and had swelling.”

Some of the young women in the sample described having to reconcile contradictory information from different sources, as in the example below:

“My mother told me to use cloth whereas my sister told me to use the sanitary napkin. I felt comfortable with a sanitary napkin. I was able to move freely in it.”

While young women often received misinformation, many participants did describe receiving menstrual supplies from female relatives without incident once they reached menarche. Most reported receiving sanitary napkins; fewer mentioned cloths.

Most participants had little knowledge of the medical or practical aspects of menstruation and believed that they may be at risk of harming others or themselves while menstruating.

### Social challenges

In the social sphere, the primary menstruation-related challenges included stigma about menstruation, limited opportunities to discuss menstruation with others, and limitations on mobility and social interactions.

#### Menstrual stigma

The majority of young women received messages from female relatives stating menstruation was dirty and impure. Very few young women participated in worship activities during menstruation. While some did not know the reason why, most reported that they were not allowed to pray or perform *puja* because they were dirty, unclean, or impure.

“My mother told me that during periods you are not clean and you cannot offer prayer and cannot sit in the area where prayers are offered.”

“We are not pure so we cannot touch.”

“I could not go into the temple during the prayers … because I was dirty”

While stigmatising messages were the most prominent type of message, a few participants reported that their mothers and other relatives told them that menstruation was natural and important for fertility:

“At first I was confused. Then I told my mom about it, so she told me that it’s natural.”

“She told me there’s nothing wrong about it.”

When these conversations occurred, young women reported that learning that menstruation is natural and normal made them feel better about menstruation:

“I felt bad initially as I had no idea about this beforehand but after that my mother explained me everything and thereafter there were no issues … She told me there is nothing to worry. You are feeling a bit different as this is the first time but this is quite natural and a monthly affair.”

Communication helped to normalise menstruation, which suggests menstruation stigma is modifiable.

#### Limited discussion of menstruation

Participants had few people to speak to about menstruation. Mothers, other female relatives, close female friends, and female teachers were presented as a narrow pool of possible confidants:

“[My mother] told me not to discuss this with anyone except her, grandmother, elder sister and my friends.”

“Nobody would come to know … I used to tell my mom. I used to tell my sister too.”

“She advised me to discuss this with my other female friends or teachers who have undergone such situation if anytime this occurs during school time.”

Only a small minority (five young women out of 70) described receiving education about menstruation from schools or NGOs, further indicating that contexts for discussing menstruation are limited.

Young women were advised against discussing menstruation with certain groups of people. Many participants had been directed that they should not speak about menstruation with boys and men, even those in their immediate family:

“[My mother] told me that I should not tell boys about it … she just said I know about it your aunts know. But don’t tell your father about it. And don’t open this [Stayfree] (sanitary pads) pack in front of your brothers.”

A subset of participants were told not to discuss menstruation with people from other households or with younger girls who had not yet menstruated:

“When the periods happened, my mother would say don’t stay anywhere you don’t have to be. I should stay clean and if I have to go to other people’s homes I should not mention everything in idle talk. My mother would say all this … If you would tell all these things to people they would consider it to be wrong.”

“She said that you should not tell anyone about your periods. I can tell my mother or my friend who have had their periods. But I can’t tell young children because they don’t know about it.”

Participants did not typically report knowing the reason for prohibitions against speaking about menstruation to certain groups of people, although a few participants were advised that speaking to a socially inappropriate person might be source of embarrassment and shame for both parties. Even when such restrictions were intended to protect participants from negative social consequences, however, they resulted in further limiting acceptable opportunities to discuss menstruation, additionally restricting young women’s sources of information about menstruation.

#### Limitations on mobility and interaction during menstruation

Young women were restricted from participating in many regular activities while they were menstruating. In addition to restrictions on worship, which were reported by most participants, a number of participants were advised not to go outside of the house while menstruating. Young women described limited social interactions when menstruating, as in the case of a young woman who said she cannot “go with my friends.” She related:

“My mother told that during periods I should stay at home and use sanitary pads, I should not go with my friends, and I should let my hair loose.”

A sizeable number of participants were also directed to limit interactions with men and/or boys during menstruation. For example, one young woman related, *“She told me whenever you get it, never go near boys too much and not to jump around much. That’s all.”*

For several young women, mobility was limited not just during menstruation, but was limited in general post menarche, because they had “grown up.” They learned at home and in the community that it was no longer appropriate for them to play or “roam” as freely as they had when they were children. As one young person describes:

“I played a lot till I was 14, then when my body started changing, I stopped playing … As it happens with our bodies, it changes with age. So, I started playing less when my periods started … They said now you have come of age, you should not play anymore. Now you have grown up so play within girls groups or go to college. And they said to not play with boys so much. That’s all I was told.”

As another young person says: *“Yes, like I could go out anywhere every day. Now I can’t go every day … Because of periods. It doesn’t feel good, and I was told not to go outside as well.”*

As illustrated by these quotations and expressed by several other participants, new restrictions on mobility after menarche and during menstruation were sometimes a cause of frustration and resentment. Very few participants in the sample described self-limiting their mobility due to pain and discomfort during menstruation or fear of staining garments; the predominating experience among participants was of externally imposed restrictions and guidelines on mobility and social interaction.

### Institutional challenges

Young women discussed institutional-level challenges primarily in the school context, as school was the main institution that they frequented. At school they faced shame, embarrassment, and a lack of infrastructure to support menstruation.

#### Shame and embarrassment

Overall, menstruation was not normalised in school. Both students and teachers made young women feel ashamed of menstruating while at school. Several young women described being embarrassed or ashamed, especially when they were teased by their male peers about menstruating.

“Boys use the same toilets in the morning so they come to know about this and it is very shameful … They make so much fun of girls … If anyone else listens to this, then it will be so embarrassing.”

Sometimes, teachers perpetuated menstrual stigma as well. In one example, a participant described a teacher saying it was shameful that a male janitor saw menstrual hygiene products:

“The person, who cleans the toilet, always complains the teacher about pads. So she has to make the girls understand but they never understood. One mistake brought shame to all and the teachers get embarrassed … Ma’am tells us that she feel embarrassed when a man told me about this.”

Thus, stigmatisation of menses was reinforced in school.

#### Dirty toilets

Young women complained that the toilets at school were very dirty, which made managing menstruation at school difficult. Toilets were described as unsanitary and possible sources of disease. As these young people describe:

“The toilets are quite dirty … I don’t feel like using them in that condition … It is so dirty that we can’t even change our stuff” (hesitating to say sanitary pads).

“If the bathroom is smelly and not clean, it will spread diseases as the flies and insects roam around.”

Lack of places for disposing sanitary pads was also cited as one reason the toilets were dirty. As seen in the following quotations:

“The toilets were very dirty there, some girls also made it messy. Our teachers always asked us to use dustbin but some girls never listened to them. Though toilets were cleaned on a regular basis but some girls used to make it really a mess.”

“The girls in the degree classes dirty the toilets a lot. They don’t use the dustbin to dispose of their napkins, they just keep it in the toilets.”

Because the toilets were so dirty, many young women reported avoiding using the toilets for the entire school day as much as possible (both during menstruation and other times). As one person described, *“We didn’t use toilets in school. We tried not to use toilets at all.”*

When they could not avoid using the school toilet, young women reported strategies like trying to use cleaner teachers’ toilets or getting friends to clean the bathroom area for them so that they could manage menstruation at school:

“There was not much problem [managing periods] until the 7th grade. After that, I used to prepare myself but sometimes I had no other option other than using the restroom for changing the sanitary napkins. Sometimes we would sneak into our teachers’ bathroom too because student’s toilet would be that dirty. Therefore, I didn’t like those things.”

Another young person said:

“Due to the untidy toilet there was a danger of getting infections. I tried to never use the toilet; I preferred to use my home toilet … if I wanted to use the toilet [at school], I would go with my friends and tell them to clean it a little so that I could use it.”

In spite of these challenges to MHM at school, only four young women reported regularly missing school during menstruation: one due to pain, two due to inadequately clean toilets, and one because her mother had asked her to stay home during that time. The majority of young women did attend school during menstruation.

## Discussion

This paper reports a number of themes relating to menstruation. The young women interviewed reported that they face numerous barriers to safe and healthy menstruation consistent with multiple levels of the socio-ecological model. At the individual level, young women were largely unaware of menstruation prior to menarche, leaving them unprepared for the experience. After menarche, they also faced gaps in knowledge and received incorrect information on MHM. On a family and social level, menstruation was stigmatised. Young women could only discuss menstruation with select individuals and found their regular mobility curtailed during menstruation. Finally, on an institutional level, young women experienced menstruation-related shame and embarrassment, and many schools had inadequate bathroom facilities.

Many of these findings are consistent with findings from other areas of India. Knowledge gaps and misconceptions about menstruation are a frequent finding.^16,17,19,20,22,36^ Stigma and taboo around menstruation in India are also prominent, with many studies reporting on negative messages about menstruation.^16,21,24,37^ Research has also found that there are limited opportunities for young women to discuss menstruation openly^3,19,21,22^ and that regular activities and mobility are limited during menstruation.^17,37,38^ Finally, structural difficulties with managing menstruation at school, like inadequate bathroom facilities without areas to dispose of pads, have also been identified in other research.^2,16,18,21,39^ Overall, menstruating young women face similar challenges in many regions and settings in India.

While this study supports many of the findings from other studies, conceptualising the challenges associated with menstruation within a socio-ecological framework is an innovative approach to this issue in an Indian context that suggests that multi-level interventions are needed to address these challenges. There is a demonstrated need for accurate information on the biology of menstruation and on MHM to dispel myths and misinformation. Given ignorance of menstruation at menarche was common in this study, education is needed in early adolescence, whether provided through schools, more robust NGO programming, or via other methods with broad community reach. While India has national programmes for sexual and reproductive health education in schools, significant barriers to its consistent implementation have been documented.^40,41^ The low exposure to school-based MHM education among participants indicates that there are likely gaps in the current approach to menstrual education in Lucknow schools.

However, while education is important, information alone is likely insufficient. Interventions also need to address social practices that communicate menstrual stigma, shame, and silence and can lead to internalised stigma around menses in young people. It is promising that destigmatising conversations communicating that menstruation was normal were useful to participants in this study. In addition to destigmatising interventions that engage girls and women, engaging boys and men as supports for menstruating girls and women is also warranted. In these life course interviews, boys and young men served primarily as a risk for the menstruating young woman to avoid, or, in some cases, as antagonists. However, previous research in India suggests that boys and young men are sympathetic to the challenges to menstruation^27^ and can be effective participants in menstruation-related interventions.^42^ Girls and woman also have material MHM needs: they require access to clean bathrooms and disposal facilities that are safe, hygienic, and private. Thus, this paper suggests multiple levels of the socio-ecological model as intervention targets and that a multi-level solution is needed to comprehensively address the challenges associated with menstruation, in order to facilitate a supportive context for problem-free menstruation experiences.

Other studies have identified availability and access to sanitary pads and the ability to adequately wash and dry reusable cloths as challenges for menstruating individuals in India.^3,17,20^ Interestingly, in this study, issues with accessing adequate hygiene supplies did not emerge as a prominent challenge, with most participants mentioning female relatives as a source of supplies. However, the interview guide focused primarily on how menstruation experiences impacted young womens’ day-to-day routines throughout their life course and did not probe deeply about methods of menstruation management specifically, so while this was not a challenge brought up by young women, it does not necessarily mean there are not access issues. Additionally, young women may not be aware if cost of sanitary supplies is an issue within the household if they are not participating in purchasing decisions.

Our study also diverges from previous research findings around school absence. Missing school during menstruation has been reported in many studies.^2,16–18,21,37,43^ In this study, school absence due to menstruation was not an issue for most participants in spite of the challenges identified around bathroom cleanliness and sanitary napkin disposal. Nonetheless, the difficult environment for menstruating young women at school should be addressed to best facilitate their educational achievement, as menstrual challenges can cause distraction and distress in school.^1,6,44^

Menstrual challenges described here, rooted in stigma and taboo, can be described as part of a larger system reinforcing the inequality of women and girls.^45–47^ As Indian scholars have commented, configuring menstruation as dirty, shameful, and secret emphasises women’s supposed inferiority or deficiency.^16,47^ Practices of seclusion and exclusion as detailed in this paper and others have a detrimental effect on women’s ability to participate equally in public life both in India and other countries.^44,45,48^ Thus, in addition to multi-level solutions, MHM interventions must be thoughtfully designed to prevent the unintentional reinforcement of mechanisms of women’s inequality.^45,49^

Indeed, menstrual hygiene has been framed as a human rights issue, implicating rights to privacy, health, education, work, and water and sanitation.^48^ Denying adequate provision for menstruating individuals to manage menstruation in work and educational settings, as well as the stigma of menstruation itself, are threats to gender equality.^48^ According to the human rights framework, the provision of MHM education and the reduction of menstrual stigma are preconditions for effectively addressing the material needs of menstruation such as disposal facilities and affordable, hygienic menstrual hygiene supplies.^48^

In the research sphere, Hennegan^50^ asserts that building a robust evidence base in MHM is necessary in order to support the human rights of menstruating individuals. This requires a greater investment of resources in MHM research both to quantify the scope of menstruation-related challenges and to conduct robust evaluations of menstruation-related interventions.^50^ Emphasising the human right to dignity, when evaluating such interventions, outcomes such as comfort and psychosocial well-being should be measured alongside education and employment outcomes.^50^ Kirk and Sommer^1^ suggest participatory research and intervention approaches that engage young women and girls as experts in intervention design to avoid unintentionally reinforcing menstrual stigma and shame. To this end, identifying community facilitators and existing resources for healthy and safe menstruation may be an additional avenue for research. Combining culturally competent, participatory intervention design with robust evaluation methods is crucial for building the evidence base for effective MHM solutions.

Some limitations of this paper must be noted. The present study examines the life experiences of young women in the urban slums of Lucknow. This study was intended to generate information as well as hypotheses about potential areas for intervention. However, findings should not be considered representative or generalisable to young women across India, though many of our findings are consistent with other research. Furthermore, because our recruitment partners were primarily local NGOs who had already had some contact with the communities, these participants may be slightly more connected to youth-focused services and interventions than those living in communities that do not work with NGOs. Overall, however, we did not find a high involvement with MHM programmes or interventions in our sample.

## Conclusion

Young women living in the slums of Lucknow, Uttar Pradesh, face challenges to healthy and safe menstruation. Our findings suggest the need to simultaneously target multiple levels of the socio-ecological model to facilitate a challenge-free menstruation experience and to support the well-being of adolescent girls and young women. Our findings further highlight the complex context that shapes menstruation experiences for young women in the urban slums of Lucknow, Uttar Pradesh and suggest several possible avenues for intervention. Educational interventions should be provided early, before menarche, and should engage both older female relatives and men and boys in the process of destigmatising menstruation. Normalising menstruation and menstrual management are important first steps to securing policies and resources that support MHM and the human rights of menstruating individuals. These policies and resources should include material interventions at the school level to ensure that bathrooms have adequate privacy and disposal facilities. Overall, creating a sustainable supportive environment for challenge-free menstruation requires multilevel, culturally appropriate interventions that emphasise well-being and comfort as well as more typical health, educational, and labour outcomes.
